# Feasibility, Safety, and Preliminary Efficacy of Median Nerve Stimulation for Cognitive Dysfunction in Patients With Acute Traumatic Brain Injury: Study Protocol for a Pilot Randomized Controlled Trial

**DOI:** 10.2196/95327

**Published:** 2026-08-14

**Authors:** Yu Deng, Guoyi Gao, Liang Wu

**Affiliations:** 1 Department of Neurosurgery, Beijing Tiantan Hospital, No. 119, South Fourth Ring West Road, Fengtai District, Beijing, China, Beijing, 100070, China, 1 18301674233

**Keywords:** median nerve stimulation, cognitive dysfunction, traumatic brain injury, neuromodulation, pilot randomized controlled trial

## Abstract

**Background:**

Currently, the treatment of cognitive dysfunction after acute traumatic brain injury (TBI) remains a challenge, and novel therapeutic methods are urgently needed. Median nerve stimulation (MNS) is a noninvasive neuromodulation technique that has recently shown positive effects in promoting recovery from coma after acute brain injury. It has been shown to improve cognition in healthy volunteers, and may be a potential therapeutic approach for cognitive dysfunction in patients with acute TBI.

**Objective:**

The primary aim of this study is to evaluate the feasibility, safety, and preliminary efficacy of MNS for cognitive dysfunction in patients with acute TBI.

**Methods:**

This study is designed as a prospective, single-center, assessor-blinded, sham-controlled, randomized pilot study. Thirty adult patients with cognitive dysfunction after TBI will be enrolled and randomized to either the stimulation or sham group in a 1:1 allocation ratio. In the stimulation group, MNS is performed on the right wrist (40 Hz, pulse width 300 µs, 20 s on/40 s off, 8 hours per day). The sham group will receive stimulation using an identical device set to deliver 0 mA. Feasibility, safety, and preliminary efficacy will be evaluated at 1 and 3 months after the injury. Feasibility and acceptability will be assessed via recruitment, adherence, retention, and participant-reported acceptability using Likert scales. These measures will be summarized descriptively, and completion rates will be calculated and reported. Safety will be primarily assessed by ascertainment of adverse events from enrollment to 3 months. Further safety assessment will examine side effects using a Likert questionnaire and the impact on peripheral hemodynamic responses. Additionally, the potential efficacy of MNS on cognitive dysfunction will be assessed using multiple cognitive scales; neuroradiological tests, including magnetic resonance imaging and magnetoencephalography; and cognitive-related serum biomarkers.

**Results:**

Ethics approval was obtained in January 2025, and the trial was registered on ClinicalTrials.gov in February 2026. Participant recruitment is scheduled to begin in July 2026, with completion anticipated in June 2027. Data analysis will commence after completion of follow-up assessments, and study findings are expected to be published in late 2027.

**Conclusions:**

This pilot trial will provide preliminary evidence on the feasibility and safety of MNS as a novel intervention for cognitive dysfunction after acute TBI. The results will guide the development of larger-scale trials and optimization of study design.

## Introduction

Traumatic brain injury (TBI), a leading cause of death and disability, is a significant global public health problem [1,2]. It can cause cognitive dysfunctions, including executive function, memory, attention, language, and visuospatial function, which seriously affects patients’ quality of life and places a substantial burden on families and society [3]. Currently, the main therapeutic methods for cognitive impairment include cognitive training, drug therapy, hyperbaric oxygen therapy, and aerobic exercise therapy [4-7]. These approaches have shown potential benefits for cognitive impairment. However, the improvement in overall cognitive function is inconsistent. Moreover, all the interventions are usually performed during the chronic stage of TBI, leading to often delayed and suboptimal therapeutic outcomes [8]. Thus, treatment options for cognitive impairment during the acute stage of TBI remain limited.

Recently, noninvasive neuromodulation techniques, including repetitive transcranial magnetic stimulation (TMS), transcranial direct current stimulation (tDCS), transcutaneous auricular vagus nerve stimulation, and median nerve stimulation (MNS), have been recognized as having promising potential in improving cognitive function in patients with cognitive impairment caused by stroke, intracerebral hemorrhage, and TBI [9-12]. Among these techniques, MNS is a simple, inexpensive, and noninvasive neuromodulation technique that has been shown to improve recovery after TBI and to hasten awakening from coma, as demonstrated in our previous study [13]. Furthermore, clinical studies have shown that it can effectively improve cognitive function in healthy individuals and enhance cognitive recovery following stroke [14]. Emerging preclinical evidence further suggests that MNS may exert neuroprotective effects after TBI by attenuating oxidative stress and ferroptosis, activating the nuclear factor erythroid 2-related factor 2 or glutathione peroxidase 4 signaling pathway, and promoting the expression of proteins associated with neuronal survival and synaptic plasticity [15]. These findings provide a plausible biological mechanism through which MNS may facilitate cognitive recovery during the early postinjury period. As the acute phase following TBI represents a critical window of enhanced neuroplasticity, initiating neuromodulatory interventions during this stage may maximize their therapeutic potential.

Despite these promising findings, clinical evidence regarding the use of MNS for cognitive dysfunction during the acute stage of TBI remains lacking. To our knowledge, no randomized controlled trial (RCT) has evaluated the feasibility, safety, or preliminary efficacy of MNS in this population. Therefore, we designed this pilot RCT to evaluate the feasibility and safety of MNS and to explore its preliminary effects on cognitive dysfunction in patients with acute TBI. In addition, serum biomarkers and magnetoencephalography (MEG) will be assessed to provide preliminary insights into the potential biological mechanisms underlying the effects of MNS. The findings of this pilot study will inform the design of a future definitive RCT.

## Methods

### Study Design

**Figure 1.**
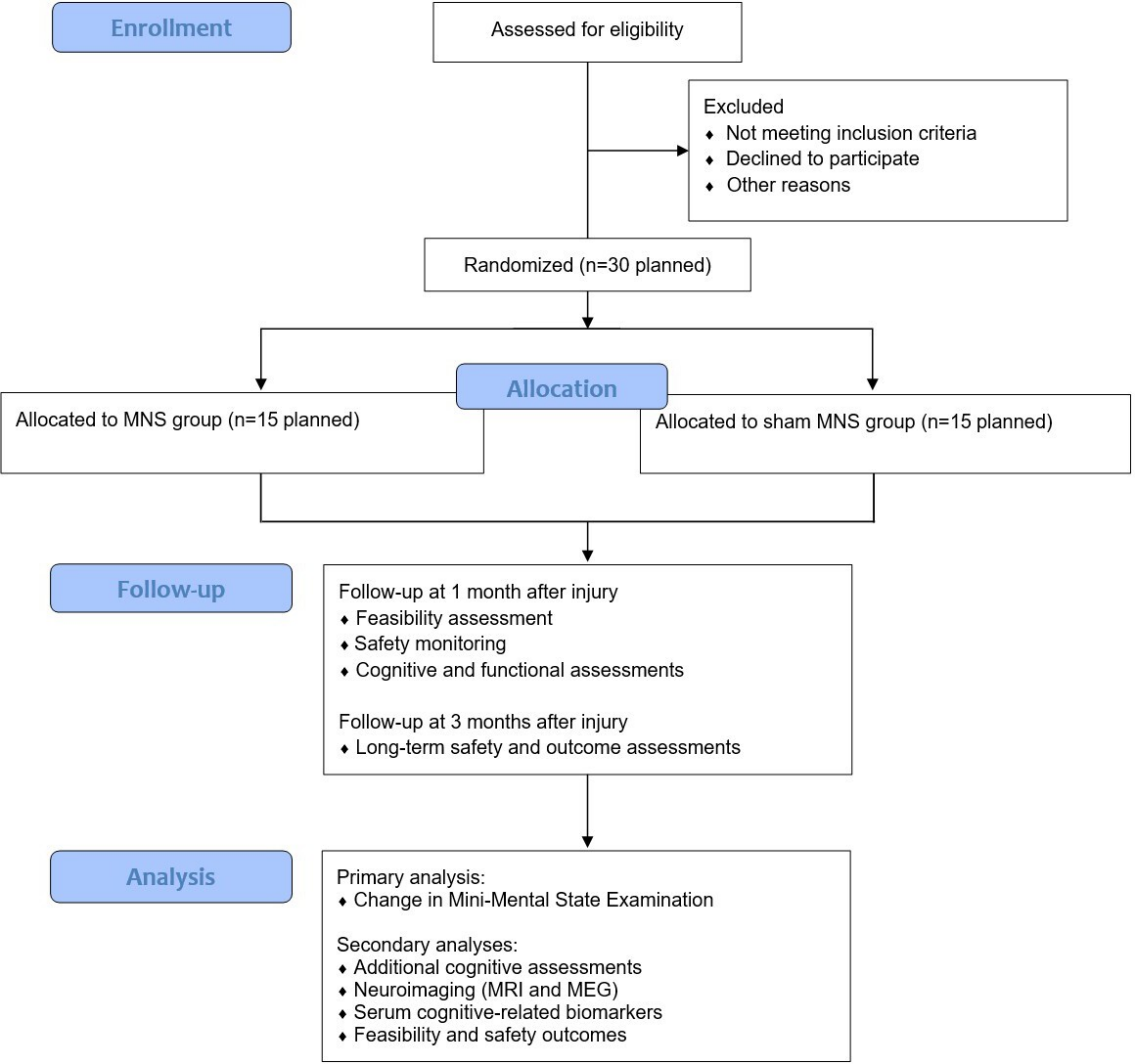
Consolidated Standards of Reporting Trials (CONSORT) flow diagram. MEG: magnetoencephalography; MNS: median nerve stimulation; MRI: magnetic resonance imaging.

This study is a prospective, single-center, assessor-blinded, sham-controlled, randomized pilot study. The study protocol was developed in accordance with the Consolidated Standards of Reporting Trials (CONSORT) extension for pilot and feasibility trials [16]. The CONSORT flow diagram is presented in Figure 1. Thirty patients with TBI and cognitive dysfunction will be enrolled and randomly allocated to an active or sham MNS group.

### Ethical Considerations

This trial was approved by the ethics commission of Beijing Tiantan Hospital, Capital Medical University (KY2024-409-02), on January 27, 2025, and is registered on ClinicalTrials.gov (NCT07413562). The accepted version of the study protocol is 3.0. The study was designed according to Good Clinical Practice, the principles of the Declaration of Helsinki, and standards for professional conduct. Written informed consent will be obtained from all participants prior to enrollment in accordance with the Declaration of Helsinki and its later amendments [17]. The results of this randomized pilot study will be disseminated via peer-reviewed publications and congress presentations. This present protocol was written based on the Standard Protocol Items for Clinical Trials.

### Participants

Patients with cognitive dysfunction after acute TBI will be asked to join the study at the Department of Neurotrauma, Beijing Tiantan Hospital. Participants will be randomly allocated in a 1:1 ratio to either the active MNS group or the sham group.

### Eligibility Criteria

The inclusion and exclusion criteria are summarized in Textbox 1.

Textbox 1. **Inclusion and exclusion criteria.**

**Inclusion criteria**

- Aged 18-64 years
- Admitted within 3 days after injury with a Glasgow Coma Scale score of 9 to 12 at admission and imaging abnormalities consistent with traumatic brain injury (TBI)
- Presence of cognitive dysfunction assessed within 1 week after injury, defined as a Mini-Mental State Examination score ≤26
- Preinjury Clinical Dementia Rating score of 0, as reported by family members
- Preinjury education ≥6 years and able to understand instructions and cooperate with cognitive assessments, magnetic resonance imaging (MRI), and magnetoencephalography (MEG) examinations

**Exclusion criteria**

- Requirement for emergent neurosurgical intervention during treatment, including surgery, intracranial pressure monitoring device placement, or drainage catheter insertion
- Unstable vital signs or hemodynamics, or unstable cardiac, pulmonary, hepatic, renal, or hematopoietic disorders
- Preexisting central nervous system disorders associated with cognitive decline, including previous TBI, intracranial infection, brain tumor, epilepsy, stroke, neurodegenerative diseases, carbon monoxide poisoning, or alcohol abuse
- Inability to complete assessments because of severe visual or hearing impairment, severe psychiatric or behavioral disorders, contraindications to MRI, or intolerance to MEG
- Short life expectancy due to critical illness
- Extensive skin lesions or scars on the right forearm, right median nerve injury, brachial plexus injury, cervical spinal cord injury, or intolerance to median nerve stimulation
- Pregnancy or lactation
- Participation in another ongoing clinical trial

The withdrawal criteria include (1) occurrence of any exclusion criterion, (2) occurrence of severe safety events, (3) poor compliance and difficulty in carrying out the trial, and (4) withdrawal of consent.

### Recruitment

Patients who are hospitalized in the Department of Neurotrauma, Beijing Tiantan Hospital, will consent to be screened for the study. A member of the research team will screen eligibility according to the criteria and take a brief medical history. The results of all screenings will be recorded both in the patients’ medical notes and on the study case report form (CRF). The potential participants will be carefully assessed for cognitive functions using the Mini-Mental State Examination (MMSE). Before enrollment, the treating investigator will assess each potential participant’s capacity to provide informed consent based on their ability to understand the study information, appreciate its implications, communicate a choice, and provide informed agreement. Participants judged to have adequate decision-making capacity will provide written informed consent themselves. If a participant is considered unable to provide valid informed consent, written informed consent will be obtained from a legally authorized representative in accordance with institutional ethics requirements. The researchers involved in the trial will be responsible for recruiting participants who have consented to participate and who meet the established inclusion criteria.

### Randomization

Once written informed consent has been obtained and eligibility has been confirmed, participants will be randomly assigned in a 1:1 ratio to either the active MNS group or the sham group. An independent investigator who is not involved in participant recruitment, intervention delivery, outcome assessment, or data analysis will generate the allocation sequence using an online computer-based randomization program. Randomization will be performed using a permuted block design with variable block sizes to ensure allocation concealment. No stratification factors will be applied. The allocation sequence will be securely maintained within the online randomization system and will remain inaccessible to the clinical investigators before randomization. After completion of the baseline assessment and confirmation of participant eligibility, the independent investigator will release the randomization code through the online system, and the participant will then be assigned to the corresponding treatment group. Clinical investigators cannot influence the randomization process.

### Interventions

Participants will receive right MNS (active or sham) for 2 consecutive weeks in addition to standard care. During the intervention period, medications that may affect cognitive function will not be permitted. The median nerve stimulator (XCH-B1; Jiangxi Nuocheng Electric Co Ltd) delivers asymmetric biphasic pulses. In the active MNS group, stimulation will be delivered at a fixed frequency of 40 Hz with a pulse width of 300 µs using a 20-second ON or 40-second OFF duty cycle. During the first treatment session, the treating investigator will set the stimulation intensity between 15 and 20 mA according to individual tolerance. The intensity may subsequently be adjusted within this range if necessary to maintain a visible thumb flexion while remaining comfortable and well tolerated by the participant. Participants in the sham group will use an identical device with the same electrode placement, treatment duration, and operating procedures as those in the active MNS group. However, the device will be programmed to deliver 0 mA, thereby providing no electrical stimulation. This sham procedure was selected to control for the nonspecific effects associated with device application while avoiding unintended activation of the median nerve. Participants will be instructed not to modify the stimulation parameters independently. Any adjustment to the stimulation intensity will be performed only by the study investigator when clinically indicated.

Two self-adhesive surface electrodes will be placed over the right median nerve on the volar aspect of the forearm. The distal electrode (anode, +) will be positioned approximately 2 fingerbreadths proximal to the wrist crease, and the proximal electrode (cathode, −) will be positioned approximately 2 fingerbreadths proximal to the distal electrode along the course of the median nerve. Before the first treatment session, study personnel will verify correct electrode placement and provide standardized instructions regarding the intervention procedure. The stimulation site will be inspected before each treatment session for erythema, irritation, blistering, or other signs of skin injury.

Stimulation will be administered for 8 hours per day throughout the 2-week intervention period by trained study personnel. If mild skin irritation or discomfort occurs, the electrodes may be repositioned, and the stimulation intensity reduced while maintaining visible thumb flexion. Stimulation will be suspended if symptoms persist and permanently discontinued if a serious device-related adverse event (AE) or clinically significant intolerance occurs, as determined by the study investigator.

Treatment adherence will be monitored by study personnel and recorded in the CRF, including the daily stimulation duration, any interruptions, device-related problems, and AEs. Device function and electrode contact will be checked before each treatment session. If device malfunction occurs, stimulation will be stopped immediately, the malfunction will be documented, and the device will be inspected or replaced before treatment is resumed. If stimulation is interrupted because of medical procedures, clinical instability, technical problems, or other unavoidable circumstances, participants will resume stimulation as soon as clinically feasible on the same day to complete the planned 8-hour daily treatment whenever possible. Missed stimulation time will not be carried forward to subsequent days. All interruptions and their reasons will be documented and incorporated into the assessment of treatment adherence.

### Study Plan

Patients with TBI are routinely screened on the seventh day after injury. If cognitive dysfunction is detected, the patient may be eligible to participate according to the inclusion and exclusion criteria. Eligible patients will be informed about the study and provided with an information leaflet. If the patient is willing to participate, an appointment will be scheduled.

#### Visit 1

At the initial visit, which will be the seventh day after injury, written informed consent will be confirmed, and the participant will then be allocated to a group. Demographic data and results of neurological examinations will be collected. Neurological function, cognitive function (via scales, MEG, and serum biomarkers), mental behavior, activity of daily living, and quality of life will be assessed. Afterward, the participant will receive a median nerve stimulator and will receive brief training and instructions. Then the stimulation phase for 2 weeks starts. The treatment will be performed in the hospital under the supervision of trained study staff. The participant is advised to contact the principal investigator during interventions if any side effects occur.

#### Visit 2 (1 Month After Injury)

At the second visit, feasibility and safety outcomes will be assessed. Participants will complete the study-specific acceptability questionnaire, and all AEs will be recorded. The stimulation statistics will be downloaded from the app, and the stimulator will be returned to the investigator. Neurological function, cognitive function, mental behavior, activity of daily living, and quality of life will be assessed again.

#### Visit 3 (3 Months After Injury)

The last visit will be 3 months after injury. In this follow-up phase, the persistence of the MNS effect will be evaluated to determine whether it is sustained or diminishes over time. At the last visit, neurological function, cognitive function, mental behavior, activity of daily living, and quality of life will be assessed for the third time, and the safety data will also be evaluated again.

### Data Protection

All data will be recorded pseudonymously, and the link between number and patient name will be stored on a separate secure clinical network server. The electronically recorded data of this study will be maintained on a password-protected database under the control of the principal investigator. All questionnaires and consent forms will be stored in a locked cabinet in a locked room requiring key card access. All additional information will be stored without any identification of group assignment on a separate database.

### Outcomes

#### Primary Outcome

##### Feasibility

Referring to the previous recommendations, the feasibility of this pilot study will be evaluated based on the following measures: (1) enrollment rate—the proportion of participants who meet the eligibility criteria and provide consent after baseline screening; (2) recruitment rate—the mean number of participants enrolled in the study per month; (3) adherence rate—the proportion of completed scheduled stimulation hours, which will be used to assess the acceptability of the interventions; and (4) retention rate—the proportion of participants who complete all outcome measurements from the baseline to follow-up assessments [15,18]. These measures will determine whether to proceed with a future main trial.

##### Safety Evaluation

Safety will be evaluated throughout the intervention period by recording all AEs, including pain, uncomfortable cutaneous sensation, dizziness, abnormal blood pressure, abnormal heart rhythm, headache, nausea, and seizures. At the completion of the intervention, participants will complete a study-specific 10-item questionnaire based on a 5-point Likert scale (Multimedia Appendix 1) to evaluate treatment acceptability and participant-reported symptoms. The questionnaire evaluates participants’ understanding of the intervention, perceived benefit, treatment comfort, willingness to receive MNS again, and potential treatment-related symptoms. Each item is scored from 1 to 5, with higher scores indicating greater agreement or symptom severity, as appropriate.

### Exploratory Outcomes

The schedule of enrollment, interventions, and assessments is summarized in Table 1 in accordance with the SPIRIT (Standard Protocol Items: Recommendations for Interventional Trials) guidelines [19]. The main exploratory cognitive outcome will be the change in MMSE score from baseline (visit 1) to 1 month after injury (visit 2). MMSE assessments will be conducted by 2 trained occupational therapists who are blinded to treatment allocation and are not involved in the intervention delivery. Secondary exploratory outcomes will include other cognitive assessments, including Montreal Cognitive Assessment, Verbal Fluency test, Boston Naming Test, Rey-Osterrieth Complex Figure Test, Rey Auditory Verbal Learning Test, Stroop test, Trail Making Test (parts A and B), Symbol Digit Modalities Test, and Digit Span Test [20,21]. Moreover, assessment of mental behavior will be assessed using the Neuropsychiatric Inventory, Pittsburgh Sleep Quality Index, Hamilton Anxiety Scale, Hamilton Depression Scale, Revised Apathy Rating Scale, and Cohen-Mansfield Agitation Inventory [22-26]. Neurological functions will be assessed by the Glasgow Coma Scale and modified Rankin Scale [27,28]. For assessment of activity of daily living and quality of life, the modified Barthel Index, Short Form 36 Health Survey, and Functional Activities Questionnaire will be evaluated. For neuroradiological assessment, functional connectivity within the default mode network and execution control network will be evaluated using MEG. Serum cognitive biomarkers including tau, amyloid beta, glial fibrillary acidic protein, S100 calcium-binding protein B, and alpha-synuclein will be tested.

**Table 1.** Schedule of enrollment, interventions, and assessments.

Time points	Enrollment	Allocation (day 7)	1 month	3 months
Enrollment				
Eligibility screen	✓			
Informed consent		✓		
Randomization		✓		
Interventions				
MNS^a^		2 weeks 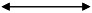 ^b^		
Sham MNS		2 weeks 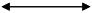		
Assessments				
Demographics, medical history	✓			
Laboratory data	✓		✓	✓
Neurological examination	✓		✓	✓
Neuroimaging (MRI^c^ and MEG^d^)		✓	✓	✓
Serum biomarkers		✓	✓	✓
Cognitive function		✓	✓	✓
Neuropsychiatric behavior		✓	✓	✓
ADL^e^ and quality of life		✓	✓	✓
Compliance			✓	✓
Drug combination	✓	✓	✓	✓
SAEs^f^ or AE^g^s			✓	✓

a MNS: median nerve stimulation.

b The intervention commenced 7 days after injury and was administered for 2 consecutive weeks.

c MRI: magnetic resonance imaging.

d MEG: magnetoencephalography.

e ADL: activities of daily living.

f SAE: serious adverse event.

g AE: adverse event.

### Sample Size

This study is designed as a pilot RCT to evaluate the feasibility and safety of right MNS and to obtain preliminary estimates of treatment effects for planning a future definitive trial. Therefore, a formal sample size calculation based on statistical power for efficacy outcomes was not performed. A total sample size of 30 participants (15 per group) was considered appropriate for this pilot study. This sample size is consistent with recommendations for pilot randomized trials and is considered sufficient to estimate key feasibility parameters, including recruitment, adherence, retention, acceptability, and safety, as well as to provide preliminary estimates of treatment effect size and variability for planning a future definitive RCT [29]. In addition, the pilot study will be used to evaluate the predefined progression criteria and determine whether protocol modifications are required before proceeding to a larger trial.

### Blinding

Investigators in charge of the recruitment and follow-up evaluation, outcome assessors, and data analysts will be blinded to group allocation. Participants and independent researchers who apply the MNS will not be blinded. Researchers who monitor patients’ safety and perform risk management can access group allocation if necessary. Before the outcome assessment begins at every follow-up evaluation, the participants will be reminded not to reveal any information about their group allocation to decrease the risk of unblinding. If the investigator can detect details of group allocation during follow-up, another blinded researcher will evaluate the outcome. To minimize performance bias, participants in both groups will receive identical standard care, follow the same treatment schedule and assessment procedures, and be managed according to the same clinical protocol throughout the study.

### Safety Monitoring

All AEs will be monitored throughout the study and recorded in the CRFs by the study investigators. Participants will be assessed for AEs during each intervention session and at every scheduled follow-up visit. The principal investigator will review all reported AEs and determine their severity, seriousness, and potential relationship to the intervention. Serious AEs will be reported promptly to the institutional ethics committee in accordance with institutional requirements. Participants will discontinue the intervention if they experience a serious device-related AE, meet any withdrawal criterion, or request withdrawal from the study.

An independent data monitoring committee (DMC) has been established to oversee participant safety, study conduct, and data integrity throughout the trial. The DMC will periodically review safety data and make recommendations regarding the continuation, modification, or termination of the trial if necessary. The study will also be conducted under the oversight of the institutional ethics committee, which will monitor compliance with the approved protocol and applicable ethical requirements.

### Data Analysis

The feasibility outcomes will be reported using descriptive statistics. Recruitment, enrollment, adherence, and retention rates will be presented with 95% CIs. AEs and their frequency will also be summarized descriptively. Participant-reported symptom and acceptability questionnaire scores will be summarized using frequencies, percentages, medians, and IQRs, as appropriate. Baseline demographic and clinical characteristics will be summarized descriptively by the treatment group. No formal statistical significance testing will be performed for baseline characteristics. Exploratory cognitive outcomes will be analyzed according to the intention-to-treat principle where applicable. Missing outcome data will be handled using multiple imputation if appropriate. As this is a pilot feasibility trial with a small sample size, efficacy analyses are exploratory and are not powered to demonstrate definitive treatment effects. Group differences will be estimated using appropriate statistical methods according to the data distribution, with emphasis placed on effect sizes and 95% CIs rather than statistical significance. No formal adjustment for multiple comparisons will be performed. The estimated effect size for the MMSE will be used to inform the sample size calculation for the future definitive RCT.

### Progression Criteria

The primary purpose of this pilot study is to determine whether a future definitive RCT is feasible. Progression to a full-scale RCT will be guided by predefined feasibility and safety criteria rather than exploratory efficacy outcomes. Specifically, progression will be considered feasible if the following criteria are met: (1) an enrollment rate of at least 60% among eligible patients; (2) a recruitment rate of at least 2 participants per month; (3) an adherence rate of at least 80% of the prescribed stimulation sessions; (4) a retention rate of at least 80% at the 3-month follow-up; (5) no unexpected serious device-related AEs attributable to the intervention; and (6) acceptable participant satisfaction with the intervention, defined as a median score of ≥4 on the 5-point Likert acceptability questionnaire. If one or more of these criteria are not achieved, the feasibility findings will be carefully reviewed to identify barriers and determine whether modifications to the study design, intervention protocol, recruitment strategy, or outcome assessments are required before proceeding to a future definitive RCT. Exploratory efficacy outcomes will be used to estimate treatment effects and variability to inform the sample size calculation and design of the subsequent trial, but they will not determine progression to the definitive study.

## Results

Ethics approval for this study was obtained in January 2025, and the trial was registered on ClinicalTrials.gov in February 2026. Participant recruitment is scheduled to begin in July 2026, with completion anticipated in June 2027. As of manuscript submission, participant recruitment has not yet commenced. Data analysis will commence after completion of the 3-month follow-up assessments, and the study findings are expected to be published in late 2027. The results of this pilot trial will provide evidence regarding the feasibility, safety, and preliminary efficacy of MNS in patients with cognitive dysfunction following acute TBI and will inform the design of a future definitive RCT.

## Discussion

### Principal Findings

This pilot RCT is expected to provide the first prospective evidence regarding the feasibility, safety, and preliminary efficacy of right MNS for cognitive dysfunction following acute TBI. We hypothesize that MNS will be feasible to implement during the early postinjury period, demonstrate an acceptable safety profile, and show promising signals of benefit in cognitive recovery, daily functioning, and quality of life. In addition to evaluating clinical outcomes, this study will explore changes in serum cognitive biomarkers and MEG, which may provide preliminary insights into the neurophysiological mechanisms underlying MNS. The anticipated findings will inform the design, outcome selection, and sample size estimation of a future definitive RCT.

Multiple noninvasive interventions of neuromodulation have been suggested to improve the cognitive function of TBI survivors in the chronic stage [9-12]. A key strength of this protocol is that it will be the first RCT to assess the feasibility, safety, and preliminary efficacy of MNS for improving cognitive dysfunction, daily functioning, and quality of life after acute TBI. In addition, serum cognitive biomarkers and MEG will be measured to help explain the neural mechanisms by which MNS improves cognitive function after TBI. We believe the findings from this pilot study will contribute to deciding whether to proceed with the forthcoming large-scale trial and, if needed, redesigning the protocol such as eligibility criteria, the dose of interventions, assessment schedule, and sample size. As a pilot study, the primary objective is to inform the design of a definitive trial rather than to draw firm conclusions regarding efficacy.

Compared with TMS and tDCS, MNS is more convenient and economical, has fewer side effects, and has been used to promote recovery from acute coma [12,13]. However, the effects of MNS in cognitive dysfunction after acute TBI have yet to be tested. In this study, we will limit inclusion to participants who have a moderate TBI event 7 days before recruitment and are clinically diagnosed as being in early cognitive dysfunction. Because no study to date has examined the effects of MNS on cognitive dysfunction after acute TBI, our study design and stimulation parameters are based on findings from patients with acute TBI-related coma. In our previous study involving participants with a traumatic coma 7 to 14 days after brain injury, right MNS stimulation 8 hours per day for 2 weeks showed clinically meaningful effects on emergence from coma, level of consciousness, and functional outcomes [13]. Therefore, the intervention is initiated in the early postinjury period and lasts for 2 weeks.

### Limitations

The present study has some potential limitations. First, the study aims to enroll patients with moderate TBI. Therefore, the findings should be interpreted with caution, particularly when generalizing to patients with mild or severe TBI. Second, because the sham intervention does not produce sensory and motor responses comparable to active MNS, participant blinding cannot be fully guaranteed, which may introduce expectancy effects and performance bias. This limitation should be considered when interpreting the study findings. Furthermore, results will only reflect short-term effects as the treatment duration will be only 2 weeks, and differences in patient lifestyles, treatment, and rehabilitation during the follow-up period may affect results. Therefore, we will collect relevant data during the follow-up period and adjust statistical analyses if needed.

### Conclusions

If the predefined feasibility criteria are met, the findings from this pilot study will support the development of a fully powered RCT to further evaluate the clinical effectiveness of MNS in patients with cognitive dysfunction following acute TBI. The study results will be disseminated through publication in peer-reviewed journals and presentations at national and international scientific conferences. Trial results will also be reported in the clinical trial registry in accordance with applicable reporting requirements.

## Supplementary material

10.2196/95327 Multimedia Appendix 1 Study-specific participant symptom and acceptability questionnaire.

10.2196/95327 Checklist 1 SPIRIT checklist.

## Abbreviations

AE: adverse event
CONSORT: Consolidated Standards of Reporting Trials
CRF: case report form
DMC: data monitoring committee
MEG: magnetoencephalography
MMSE: Mini-Mental State Examination
MNS: median nerve stimulation
RCT: randomized controlled trial
SPIRIT: Standard Protocol Items: Recommendations for Interventional Trials
TBI: traumatic brain injury
tDCS: transcranial direct current stimulation
TMS: transcranial magnetic stimulation
