# Supplementary material for: Feasibility, Safety, and Preliminary Efficacy of Median Nerve Stimulation for Cognitive Dysfunction in Patients With Acute Traumatic Brain Injury: Study Protocol for a Pilot Randomized Controlled Trial
Source: JMIR Res Protoc. 2026 Aug 14;15:e95327. doi: 10.2196/95327 (PMC13475781; doi:10.2196/95327)
Supplement: Supplementary file 1 [file resprot-v15-e95327-s001.docx]

**Multimedia Appendix 1.** Study-specific participant symptom and acceptability questionnaire. Please indicate the extent to which you agree with each statement using the following 5-point. Likert scale: 5 = strongly agree, 4 = agree, 3 = neither agree nor disagree, 2 = disagree, 1 = strongly disagree.

| **Item** | **Score** |
| --- | --- |
| 1. I received sufficient information about the MNS treatment procedure. |  |
| 2. I believe the MNS treatment improved my symptoms. |  |
| 3. I felt comfortable during the MNS treatment. |  |
| 4. I would be willing to receive MNS again in the future if needed. |  |
| 5. I experienced pain during the MNS treatment. |  |
| 6. I experienced palpitations during the MNS treatment. |  |
| 7. I experienced skin discomfort during the MNS treatment. |  |
| 8. I experienced headache or nausea during the MNS treatment. |  |
| 9. I experienced seizures during the MNS treatment. |  |
| 10. I experienced any other discomfort during the MNS treatment. |  |
